# Effect of a web-based intervention on family sex education for preschool children’s parents: a cluster randomized trial

**DOI:** 10.3389/fpubh.2026.1817229

**Published:** 2026-04-13

**Authors:** Zhao Chen, Rong Zhang, Yiru Wang, Yongli Li, Ying Liao, Yingling Zhang, Maoxu Liao

**Affiliations:** 1 School of Public Health, Southwest Medical University, Luzhou, China; 2 Information and Education Technology Center, Southwest Medical University, Luzhou, China

**Keywords:** family sex education, intervention study, online education intervention, parents of young children, theory of planned behavior

## Abstract

**Background:**

Early childhood represents a critical period for sex education and family-based education is essential to this process. However, many parents lack the knowledge and skills to deliver effective sex education, which significantly hinders implementation and effectiveness for this age group.

**Methods:**

In a cluster-randomized trial conducted in 2022, parents of preschool children from four kindergartens in Luzhou City were assigned by class to receive either a 6-month online sex education program grounded in the Theory of Planned Behavior (TPB) via WeChat or conventional health education. Parental knowledge, attitudes, and practices (KAP) were assessed at baseline and post-intervention. Intervention effects were evaluated using Analysis of Covariance (ANCOVA) and Generalized Estimating Equations (GEE) to account for baseline covariates and potential cluster effects.

**Results:**

A total of 217 parents were enrolled in the study. Post-intervention, the intervention group demonstrated significantly higher scores and improved pass and good rates across all KAP (knowledge, attitudes, and practices) dimensions compared to the control group (*p* < 0.05). Notably, the KAP consistency rate and parental preference for online learning channels also increased significantly in the intervention group (*p* = 0.001).

**Conclusion:**

The TPB-based online intervention effectively enhances parental competencies in early childhood sex education. This digital model provides a scalable and accessible strategy for health promotion, with significant implications for narrowing the implementation gap in family-based sex education and supporting children’s long-term sexual health.

## 1 Background

Sex education for children is a major global public health concern. However, its implementation often encounters significant barriers—as seen in India, where programs are stalled by lack of policy support and social stigma (1) and in China, where insufficient teacher training and inadequate teaching materials result in only 37% of schools offering related content (2). Inadequate sex education increases the risk of sexual abuse (3), which affects 12–13% of children globally (4). Associated health and behavioral consequences include depression (5), early sexual debut (6), multiple sexual partners (7), and post-traumatic stress disorder (PTSD) (8). Preschoolers, due to their low safety awareness, are particularly vulnerable and require increased attention to reduce these long-term risks.

Sex education for children relies on school- and family-based approaches. For preschoolers, parent-led education within the family is primary. Parents act as the first educators of sexual knowledge and safety. Effective early childhood family sex education (EFSE) reduces risk of child sexual abuse, improves later school-based CSE effectiveness, delays sexual debut, and lowers sexually transmitted infections incidence (9–13). However, EFSE quality depends heavily on parental knowledge, attitudes, and practices (14). Many parents struggle due to negative attitudes, limited knowledge, and low self-efficacy (15–17). Therefore, identifying effective strategies to enhance parents’ knowledge, attitudes, and practices related to sex education is essential for promoting children’s healthy sexual development and overall sexual health.

In 2018, the United Nations Educational, Scientific and Cultural Organization (UNESCO) and the United Nations Population Fund (UNFPA) advocated comprehensive sexuality education (CSE) by prioritizing gender equality in sexual health, gender identity, and sex education (1, 18). CSE has proven effective in improving sexual health literacy, reducing human immunodeficiency virus (HIV) risk, and delaying sexual debut (19–21). Recent studies have explored diverse enhancement methods, such as group training (22), classroom activities (23), and parenting classes (24). However, family sex education in China faces significant cultural barriers. Deeply influenced by traditional Confucianism, sexuality remains a taboo topic (25), making conventional resources like public lectures and courses difficult to access. To bypass these constraints, digital media offers a promising and discreet intervention pathway, which has been proven by recent reports to enhance sexual knowledge and improve parent–child communication (26, 27). This approach is highly suitable for China, where extensive internet (74.4%) and mobile (99.6%) penetration strongly support scalable online education (28).

Yet, translating this technological potential into practical application remains a challenge. Although international guidelines, including the International Technical Guidance on Sexuality Education (ITGSE) and the National Sexuality Education Standards (NSES), emphasize the necessity of early childhood sex education (18, 29), and China has enacted supportive legislation (30–32), a substantial implementation gap remains. Current research in China focuses mainly on adolescents and young adults (33, 34), employing largely cross-sectional designs (35), with few online interventions targeting parents of young children. In response, based on prior baseline surveys of a parent-targeted preschooler family sex education (PFSE) program (36) by the Southwest Medical University Student Sex Education Team, which indicated that parental knowledge and attitudes shape educational practices with the internet being a common source for acquiring sex education knowledge (37), this study implemented China’s first online sex education intervention for this group.

To ensure a systematic design and robust evaluation, this intervention was guided by the Theory of Planned Behavior (TPB) (38, 39), which posits that behavior is influenced by attitudes, subjective norms, and perceived behavioral control, this study utilized a validated scale (36) to fulfill its primary objective. Specifically, this study aimed to evaluate the effectiveness of a web-based educational intervention grounded in the Theory of Planned Behavior in improving parents’ knowledge, attitudes, and practices regarding early childhood sex education. Furthermore, this approach allowed for the assessment of current parental KAP status and the feasibility of such interventions in the Chinese context.

## 2 Participants and methods

### 2.1 Participants and sampling methods

Children in China attend kindergarten from ages 3 to 6, typically divided into junior (3, 4), middle (4, 5), and senior (5, 6) classes, with educational content adapted to each group’s cognitive abilities (36). This project was led by the early childhood sex education team at Southwest Medical University in Luzhou, China. Following a baseline survey in 2021 (36), two public and two private urban kindergartens (each with >150 students) were selected. From these, approximately 50–60 children per kindergarten were randomly chosen by class group, and their parents were included as participants. Participants were assigned using cluster randomization (unit: class) with a sequence generated via SPSS 24.0 (IBM Corp., Armonk, NY, USA). Allocation concealment was maintained by an independent researcher using opaque, sealed envelopes, which were opened only after baseline assessments were completed and participants were formally enrolled. To minimize cross-contamination, kindergartens were geographically separated. This study received approval from the Ethics Committee of the Affiliated Hospital of Southwest Medical University (KY2021280). Written informed consent was obtained from the participants. All methods were carried out in accordance with the ethical principles of the Declaration of Helsinki 1964.

### 2.2 Sample size calculation

The sample size was estimated using G*Power software (version 3.1.9.7; Heinrich Heine University Düsseldorf, Düsseldorf, Germany) for an independent *t*-test. To detect a medium effect size (Cohen’s *d* = 0.5) with a statistical power of 0.80 and a significance level of *α* = 0.05 (two-tailed), a base sample of 128 participants was initially required. To account for the cluster randomized design, a design effect of 1.6 was applied based on previous relevant studies (estimated ICC = 0.011) (40, 41), resulting in a total required sample of 205 participants.

### 2.3 Survey content

Based on extensive reference to relevant research, the study employed a self-designed “Questionnaire on Knowledge, Beliefs, and Behavioral Status Related to Sexuality Education in Early Childhood (parent’s version)”. The questionnaire demonstrated high internal consistency, with an overall Cronbach’s *α* of 0.801 and sub-dimension α values of 0.726, 0.805, and 0.898 for knowledge, attitudes, and practices, respectively. Content and construct validity were established through expert panel review and factor analysis in prior validation studies (36). This scale has been successfully applied in our team’s previous baseline survey for the PFSE project (36), further confirming its reliability and validity for the target population. The survey content encompassed the following: (1) The basic demographic characteristics of the preschool children and their parents, including the child’s gender, ethnicity, sibling status, left-behind status, the highest education attained by father and mother, the child’s primary caregiver, and the family’s average annual income; (2) the knowledge level was assessed using a 9-item questionnaire, with each correct answer assigned one point (total score 0–9). A score >6 (60%) was defined as the pass level and >7 (75%) as the good level; (3) the attitude was measured using an 8-item 5-point Likert scale (total score 0–40), with a score >24 (60%) considered as the pass level and >30 (75%) as positive level; (4) the practice was evaluated using an 11-item questionnaire on education frequency, scored 0–3 per item (total score 0–33). A score >20 (60%) indicated pass level and >25 (75%) good level; (5) KAP consistency status, which was defined as simultaneously meeting the criteria for good knowledge, positive attitude, and good practices, with the rate calculated as the proportion of participants meeting all three criteria within the study population.

### 2.4 Survey methods

Two questionnaire surveys (pre- and post-intervention) were administered over a 6-month period. For the control group, both baseline and final surveys were conducted face-to-face by trained university students. For the intervention group, online surveys were used: during baseline data collection, participants were instructed to follow the official WeChat account, after which a QR code was immediately provided to complete the baseline questionnaire. A second QR code was distributed via the same platform at the end of the intervention to complete the post-intervention survey using the same questionnaire.

### 2.5 Intervention methods

A 6-month online education intervention was delivered to the intervention group following the baseline survey. Parents in the intervention group were instructed to subscribe to a WeChat official account (*YouErXingZhiShi*), developed and updated regularly by the research team with sex education content. The control group received only general health education. The intervention program was comprised of the following four thematic modules, each of which was comprised of specific content: physical health; life education; safety education; and gender education. The detailed content and delivery frequency of online materials are presented in Table 1.

**Table 1:** Thematic modules, learning content, and delivery frequency of the online sex education intervention for parents.

Education theme	Specific aspects	Learning content	Intervention method and frequency
Physical health education	Basic body composition and reproductive organs Sexual hygiene habits related to health and body appreciation	Basic knowledge of body composition; relevant knowledge of important organs in the body; basic knowledge and differences between male and female reproductive organs; sexual hygiene habits related to health; love for the body.	Picture Books: 1 time/week; Videos: 1 time/month; Popular Science Articles: 1 time/week.
Life education	Birth of life, pregnancy and birth of babies, family functions, etc.	Birth of animals; origin of human life; process of fetal growth in the mother’s womb; basic knowledge of the placenta; ways of fetal birth; different types of families; family functions; learning gratitude.	
Safety education	Recognizing private parts, refusing unwanted physical contact, learning self-protection, etc.	Basic knowledge of private parts; protection of private parts; knowledge about body contact; distinguishing good/bad body contact; coping strategies for good/bad body contact; how to seek help.	
Gender education	Appearance of boys and girls, diverse interests and hobbies of boys and girls, beautiful ideals, etc.	Physiological differences between boys and girls; recognition of differences in appearance between boys and girls; respecting others’ and one’s own appearance; differences in interests and hobbies between boys and girls; respecting oneself and others; discussing personal ideals; professions pursued by boys and girls; how to achieve personal ideals.	

### 2.6 Statistical analysis

After the offline questionnaire was collected, data underwent manual review with double entry using EpiData (version 3.1; EpiData Association, Odense, Denmark). Data management and descriptive statistics were conducted using SPSS 24.0 (IBM Corp., Armonk, NY, USA), while advanced statistical modeling was performed using R software (version 4.3.2; R Foundation for Statistical Computing, Vienna, Austria).

At baseline, independent-samples *t*-tests (for continuous data) and chi-square (*χ^2^*) tests (for categorical data) were employed to compare demographic characteristics and baseline Knowledge, Attitude, and Practices (KAP) scores between the intervention and control groups, thereby assessing the balance of randomization.

To evaluate the intervention effects, Analysis of Covariance (ANCOVA) was utilized to examine post-intervention differences between the groups for continuous outcome variables (i.e., KAP scores). Individual baseline scores were included as covariates to control for initial variations and accurately isolate the net effect of the online educational intervention. The ANCOVA results were reported as estimated marginal means ± standard error (mean ± SE) adjusted for baseline differences, with partial eta squared ( $\eta_{p}^{2}$ ) calculated to determine the effect size of the intervention. For categorical variables, post-intervention between-group differences were analyzed using chi-square (*χ^2^*) tests.

Furthermore, given the cluster-randomized design involving four kindergartens, a sensitivity analysis was conducted using Generalized Estimating Equations (GEE) with an exchangeable correlation structure. This approach was adopted to account for potential clustering effects and to verify the robustness of the primary findings. All statistical tests were two-sided, with the level of significance set at *α* = 0.05.

## 3 Result

### 3.1 Participant flow and baseline characteristics

**Figure 1:**
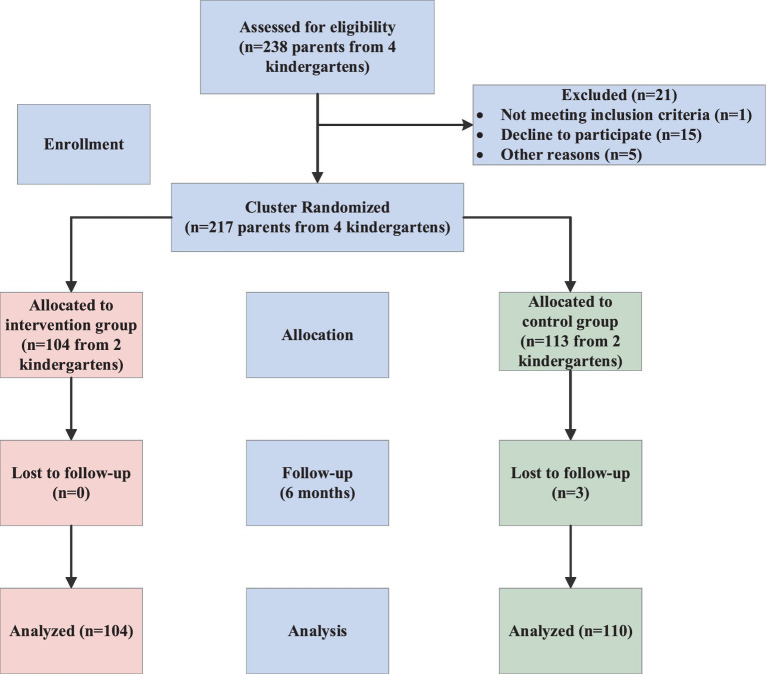
CONSORT flow diagram of participant recruitment, randomization, and follow-up in the trial.

A total of 217 parents participated in the baseline survey, with 104 assigned to the intervention group and 113 to the control group. The detailed flow of participants through the trial, including randomization and follow-up, is presented in the CONSORT diagram (Figure 1). Comparative analysis demonstrated no statistically significant differences between the two groups in child gender, ethnicity, sibling status, left-behind status, annual family income, parental ages, paternal and maternal education levels, or primary caregiver (*p* > 0.05), indicating that the groups were balanced and comparable at baseline (Table 2).

**Table 2:** Comparison of baseline demographic characteristics between the intervention and control groups of parents [*n(%)/* $\bar{\chi }\pm S$ ].

Comparison items	Classification	Total sample (*n* = 217)	Intervention group (*n* = 104)	Control group (*n* = 113)	*t/χ^2^*	*p*
Child’s gender	Male	101 (46.54)	45 (43.27)	56 (49.56)	0.861	0.414
	Female	116 (53.46)	59 (56.73)	57 (50.44)		
Ethnicity	Han ethnicity	215 (99.08)	104 (100.00)	111 (98.20)	1.858	0.499
	Others	2 (0.92)	0 (0.00)	2 (1.80)		
Residence location	Rural area	72 (33.18)	36 (34.62)	36 (31.86)	5.838	0.06
	Township	62 (28.57)	22 (21.15)	40 (35.40)		
	Urban area	83 (38.25)	46 (44.23)	37 (32.74)		
Only child	Yes	58 (73.27)	26 (75.00)	32 (28.32)	0.305	0.646
	No	159 (26.73)	78 (25.00)	81 (71.68)		
Left-behind child	Yes	86 (60.37)	36 (34.62)	50 (44.25)	2.100	0.166
	No	131 (39.63)	68 (65.38)	63 (55.75)		
Annual family income (in 10,000 RMB)	<5	110 (50.69)	50 (48.08)	60 (53.10)	2.644	0.267
	5–10	51 (23.50)	22 (21.15)	29 (25.66)		
	>10	56 (25.81)	32 (30.77)	24 (21.24)		
Parent age (years)	<30	85 (39.17)	39 (37.50)	46 (40.71)	1.463	0.691
	30–40	99 (45.62)	46 (44.23)	53 (46.90)		
	40–50	21 (9.68)	12 (11.54)	9 (7.96)		
	>50	12 (5.53)	7 (6.73)	5 (4.42)		
Father’s education level	Primary school or below	14 (6.45)	9 (8.65)	5 (4.42)	4.581	0.205
	Junior high school	84 (38.71)	35 (33.65)	49 (43.36)		
	Senior high school/vocational school	66 (30.41)	30 (28.85)	36 (31.86)		
	Associate degree/bachelor’s degree or higher	53 (24.42)	30 (28.85)	23 (20.36)		
Mother’s education level	Primary school or below	10 (4.63)	5 (4.85)	5 (4.42)	3.803	0.284
	Junior high school	81 (37.50)	32 (31.07)	49 (43.36)		
	Senior high school/vocational school	54 (25.00)	27 (26.21)	27 (23.89)		
	Associate degree/bachelor’s degree or higher	71 (32.87)	39 (37.86)	32 (28.32)		
Primary caregiver	Father	92 (42.40)	42 (40.38)	50 (44.25)	0.337	0.854
	Mother	56 (25.81)	28 (26.92)	28 (24.78)		
	Grandparents (paternal or maternal)	69 (31.79)	34 (32.69)	35 (30.97)		

### 3.2 Comparison of baseline knowledge, attitude, and practices between groups

The baseline survey of 217 parents revealed an average knowledge score of 5.01 ± 1.03 (out of 9), with passing and good rates of 31.80 and 5.07%. The average attitude score was 29.37 ± 4.75, with passing and positive rates of 90.32 and 52.53%. The average practice score was 22.99 ± 6.29, with passing and good rates of 71.89 and 48.39%. The overall KAP consistency rate was 10.06%. No significant differences were observed between the intervention and control groups in these indicators (*p* > 0.05), confirming baseline comparability (Table 3).

**Table 3:** Comparison of baseline knowledge, attitudes, and practices (KAP) regarding early childhood sex education between the two groups [*n(%)/* $\bar{\chi }\pm S$ ].

Comparison items	Classification	Total sample (*n* = 217)	Intervention group (*n* = 104)	Control group (*n* = 113)	*t/χ^2^*	*p*
Knowledge score		5.01 ± 1.03	5.09 ± 1.09	4.92 ± 0.97	1.186	0.237
Knowledge pass status	Yes	69 (31.80)	39 (37.50)	30 (26.55)	2.995	0.108
	No	148 (68.20)	65 (62.50)	83 (73.45)		
Knowledge good status	Yes	11 (5.07)	5 (4.81)	6 (5.31)	0.028	0.977
	No	206 (94.93)	99 (95.19)	107 (94.69)		
Attitude score		29.37 ± 4.75	29.60 ± 5.28	29.17 ± 4.21	0.663	0.508
Attitude pass status	Yes	196 (90.32)	93 (89.42)	103 (91.15)	0.185	0.819
	No	21 (9.68)	11 (10.58)	10 (8.85)		
Attitude positive status	Yes	114 (52.53)	60 (57.69)	54 (74.79)	2.131	0.144
	No	103 (47.47)	44 (42.31)	59 (52.21)		
Practice score		22.99 ± 6.29	23.72 ± 6.19	22.32 ± 6.34	1.646	0.101
Practice pass status	Yes	156 (71.89)	80 (76.92)	76 (67.26)	0.114	0.132
	No	61 (28.11)	24 (23.08)	37 (32.74)		
Practice good status	Yes	105 (48.39)	53 (50.96)	52 (46.02)	0.530	0.498
	No	112 (51.61)	51 (49.04)	61 (53.98)		
KAP consistency status	Yes	23 (10.60)	12 (11.54)	11 (9.73)	0.186	0.826
	No	194 (89.40)	92 (88.46)	102 (90.27)		

### 3.3 Within-group changes in knowledge, attitude, and practices before and after intervention

The within-group comparisons before and after the intervention revealed that the online intervention significantly enhanced the parents’ KAP levels. In the intervention group (*n* = 104), with the exception of the practice pass rate which did not reach statistical significance (*p* = 0.164), the parents’ knowledge scores (6.18 ± 0.89 vs. 5.09 ± 1.09), attitude scores (32.17 ± 4.00 vs. 29.60 ± 5.28), and practice scores (26.37 ± 5.71 vs. 23.72 ± 6.19) all demonstrated significant improvements compared to the pre-test (all *p* < 0.01). Additionally, the knowledge pass rate, attitude pass rate, and KAP consistency rate in the intervention group also increased significantly post-intervention (all *p* < 0.01).

In contrast, the control group (*n* = 110) exhibited no significant improvements across the measured indicators during the follow-up period. Differences in their knowledge pass rate, attitude score, attitude pass rate, practice pass rate, and KAP consistency rate between the pre-test and post-test were not statistically significant (all *p* > 0.05). Notably, the knowledge and practice scores of the control group not only failed to improve at follow-up but instead showed a slight decline compared to the pre-test (both *p* < 0.05). Details are provided in Table 4.

**Table 4:** Within-group comparison of knowledge, attitude, and practices between pre-test and post-test.

Group	Comparison items	Category	Pre-test	Post-test	*t/ χ^2^*	*p*
Intervention group (*n* = 104)	Knowledge score		5.09 ± 1.09	6.18 ± 0.89	19.738	<0.001
	Knowledge pass	Yes	39 (37.50)	82 (78.85)	41.023	<0.001
	Attitude score		29.60 ± 5.28	32.17 ± 4.00	3.860	<0.001
	Attitude pass	Yes	93 (89.42)	103 (99.04)	6.750	0.009
	practice score		23.72 ± 6.19	26.37 ± 5.71	3.213	0.002
	Practice pass	Yes	80 (76.92)	89 (85.58)	1.939	0.164
	KAP consistency	Yes	12 (11.54)	49 (47.12)	27.574	<0.001
Control group (*n* = 110)	Knowledge score		4.86 ± 0.92	4.36 ± 1.35	6.765	<0.001
	Knowledge pass	Yes	27 (24.55)	34 (30.91)	1.091	0.296
	Attitude score		29.16 ± 4.16	29.09 ± 4.88	0.117	0.907
	Attitude pass	Yes	101 (91.82)	96 (87.27)	0.762	0.383
	Practice score		22.33 ± 6.19	20.32 ± 6.92	2.394	0.018
	Practice pass	Yes	74 (67.27)	65 (59.09)	1.362	0.243
	KAP consistency	Yes	10 (9.09)	8 (7.27)	0.083	0.773

### 3.4 Post-intervention comparison of knowledge, attitude, and practices between groups

**Figure 2:**
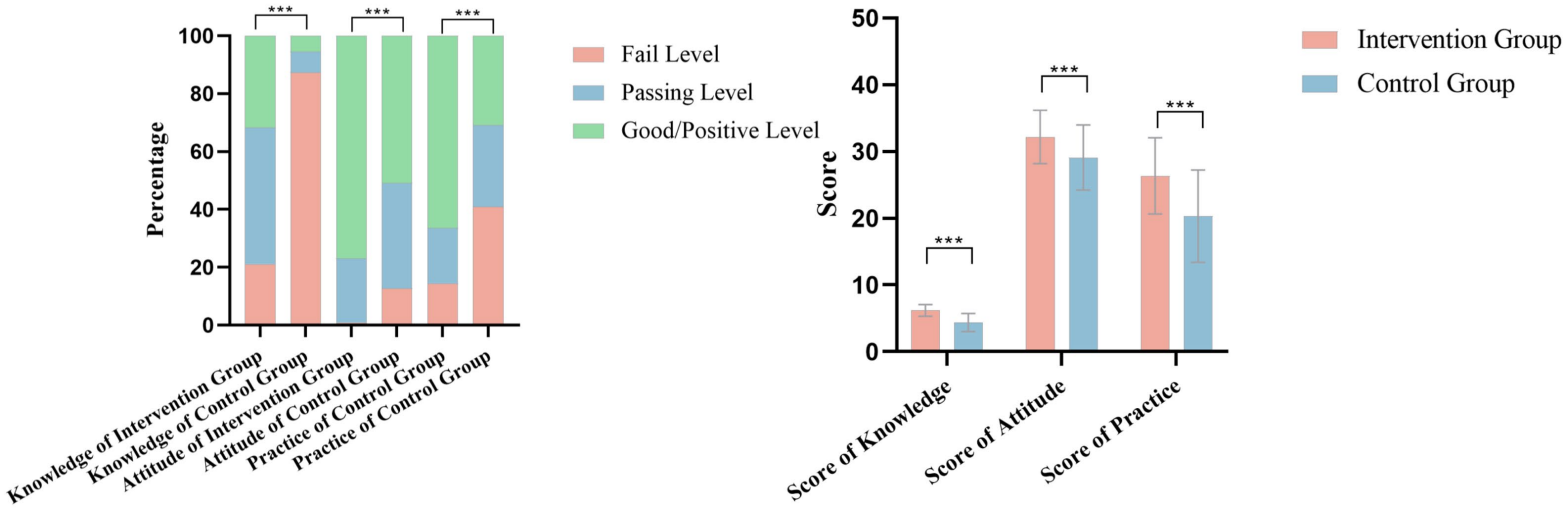
Comparison of post-intervention knowledge, attitude, and practice (KAP) scores regarding early childhood sex education between the intervention and control groups. ***p < 0.001.

The final post-intervention analysis included valid data from 214 parents (104 in the intervention group and 110 in the control group, with 3 participants lost to follow-up in the control group). The knowledge pass rates for the intervention and control groups were 78.85 and 30.91%, respectively, while the knowledge good rates were 31.73 and 5.45%; both rates were significantly higher in the intervention group (*p* < 0.05). The attitude pass rates were 99.04 and 87.27%, and the attitude positive rates were 76.92 and 50.91%, respectively; again, both were significantly higher in the intervention group (*p* < 0.05). Similarly, the practice pass rates were 85.58 and 59.09%, and the practice good rates were 66.35 and 40.00%, respectively, showing significantly higher proportions in the intervention group (*p* < 0.05). Furthermore, the KAP consistency rate was significantly higher in the intervention group compared to the control group (47.12% vs. 7.27%, *p* < 0.05). Detailed results are presented in Table 5 and Figure 2.

**Table 5:** Post-intervention comparison of knowledge, attitude, and practices between the intervention and control groups [*n*(%)/ ${\bar{\chi }}_{\text{adjust}}\pm S$ ].

Comparison items	Classification	Total sample (*n* = 214)	Intervention group (*n* = 104)	Control group (*n* = 110)	*F/χ^2^*	*p*	Effect size
Knowledge score			6.08 ± 0.07	4.46 ± 0.06^a^	297.994	<0.001	0.59 (0.51, 0.65)
Knowledge pass status	Yes	96 (44.86)	82 (78.85)	34 (30.91)	49.489	<0.001	8.33 (4.45, 15.60)
	No	118 (55.14)	22 (21.15)	76 (69.09)			
Knowledge good status	Yes	39 (18.22)	33 (31.73)	6 (5.45)	24.766	<0.001	8.06 (3.20, 20.30)
	No	175 (81.78)	71 (68.27)	104 (94.55)			
Attitude score			32.20 ± 0.44	29.10 ± 0.43^a^	25.61	<0.001	0.11 (0.05, 0.18)
Attitude pass status	Yes	199 (92.99)	103 (99.04)	96 (87.27)	11.354	<0.001	15.02 (1.92, 17.42)
	No	15 (7.01)	1 (0.96)	14 (12.73)			
Attitude positive status	Yes	136 (63.55)	80 (76.92)	56 (50.91)	15.618	<0.001	3.21 (1.76, 5.86)
	No	78 (36.45)	24 (23.08)	54 (49.09)			
Practice score			26.30 ± 0.63	20.40 ± 0.61^a^	46.33	<0.001	0.18 (0.10, 0.26)
Practice pass status	Yes	154 (71.96)	89 (85.58)	65 (59.09)	18.587	<0.001	4.11 (2.08, 8.11)
	No	60 (28.04)	15 (14.42)	45 (40.91)			
Practice good status	Yes	103 (48.13)	69 (66.35)	44 (40.00)	14.88	<0.001	2.95 (1.68, 5.18)
	No	111 (51.87)	35 (33.65)	66 (66.00)			
KAP consistency status	Yes	57 (26.64)	49 (47.12)	8 (7.27)	43.427	<0.001	11.35 (5.04, 25.56)
	No	157 (73.36)	55 (52.88)	102 (92.73)			

a Adjusted means ± standard error (mean ± SE).

Effect size (95% CI): For continuous variables (scores), effect size is partial eta squared ( $\eta_{p}^{2}$ ); for categorical variables (status), effect size is odds ratio (OR). All 95% confidence intervals correspond to their respective effect size.

Regarding the main effect analysis of KAP scores, Analysis of Covariance (ANCOVA) was employed, adjusting for individual baseline scores. The results demonstrated that parents in the intervention group achieved significant improvements across all domains. Specifically, the adjusted knowledge score in the intervention group (6.08 ± 0.07) was significantly higher than that in the control group (4.46 ± 0.06) (*F* = 297.994, *p* < 0.001, $\eta_{p}^{2}$ = 0.59). Consistent with this, the intervention group significantly outperformed the control group in both adjusted attitude scores (32.20 ± 0.44 vs. 29.10 ± 0.43, *F* = 25.61, *p* < 0.001, $\eta_{p}^{2}$ = 0.11) and adjusted practice scores (26.30 ± 0.63 vs. 20.40 ± 0.61, *F* = 46.33, *p* < 0.001, $\eta_{p}^{2}$ = 0.18) (see Table 5).

Considering the cluster-randomized design involving four kindergartens, Generalized Estimating Equations (GEE) were further applied to verify the robustness of the continuous outcome variables, thereby avoiding overestimation of the intervention effects and controlling for potential clustering. The model incorporated “kindergarten location” as the cluster variable and fitted an exchangeable correlation structure. The results indicated that, after isolating potential cluster effect interference, the improvement effect of the intervention on parents’ knowledge (Wald *χ^2^*= 1097.53, *p* < 0.001), attitude (Wald *χ^2^*= 81.79, *p* < 0.001), and practice scores (Wald *χ^2^*= 147.41, *p* < 0.001) remained statistically significant. Notably, the intra-cluster correlation coefficients (i.e., GEE *α* values) estimated by the model for knowledge (*α* = 0.015), attitude (*α* = 0.012), and practices (*α* = 0.010) all approached zero. This suggests that the cluster aggregation effect introduced by specific kindergarten environments was extremely weak and did not interfere with the intervention outcomes; the improvements in parents’ KAP levels were primarily driven by the online educational intervention itself (see Table 6).

**Table 6:** Sensitivity analysis of the intervention effect using generalized estimating equations (GEE).

Outcomes	Control group ${\bar{\chi }}_{\text{adjust}}\pm S_{\text{Robust}}$	Intervention group ${\bar{\chi }}_{\text{adjust}}\pm S_{\text{Robust}}$	Wald *χ^2^*	*p*	*α*
Knowledge score	4.470 ± 0.015	6.080 ± 0.042	1097.53	<0.001	0.015
Attitude score	29.100 ± 0.028	32.200 ± 0.357	81.79	<0.001	0.012
Practice score	20.400 ± 0.522	26.300 ± 0.117	147.41	<0.001	0.01

### 3.5 Changes in preferences for sex education information sources

In addition, parents in the intervention group were surveyed at baseline and post-intervention regarding their preferred sources of knowledge on sex education for preschool children. At baseline, the top three preferred sources were school education (72.11%), online expert lectures and training (56.73%), and books on sexual education (50.96%). After the 6-month intervention, the top three preferences shifted to online platforms (81.73%), online expert lectures and training (65.38%), and school education (63.46%). The proportion of parents preferring online channels increased significantly from 45.19 to 81.73% (*χ^2^* = 10.500, *p* = 0.001), indicating that after the online education intervention, the participants became more inclined to obtain knowledge on sex education for preschool children through online channels (Table 7).

**Table 7:** Preferences for sources of information on sexual education for preschool children among parents in the intervention group [*n* (%)].

Source and channel	Before intervention	After intervention	*χ^2^*	*p*
Parents	32 (30.77)	28 (26.92)	0.225	0.635
School education	75 (72.11)	66 (63.46)	1.488	0.222
Friends and partners	20 (19.23)	21 (20.19)	0.012	0.889
Expert lectures and training (online)	59 (56.73)	68 (65.38)	1.561	0.212
Expert lectures and training (offline)	44 (42.31)	44 (42.31)	0.185	0.901
internet	47 (45.19)	85 (81.73)	10.500	0.001
Films and TV shows	20 (19.23)	18 (17.31)	0.033	0.855
Books on sexual education	53 (50.96)	56 (53.85)	0.075	0.784
Journals and magazines	28 (26.92)	15 (14.42)	4.114	0.043

## 4 Discussion

The current study enrolled parents of preschool children. Randomized group assignment was used and baseline comparisons ensured group balance. The results demonstrated that the online intervention significantly increased parents’ family sex education knowledge, improved sex education attitudes, and promoted implementation of sex education at home. Under the framework of the TPB (38), parents’ engagement in home-based sex education practices is explained by three core constructs: behavioral attitude; subjective norm; and perceived behavioral control. The current study demonstrated that the online intervention model not only enhances parents’ knowledge and attitudes regarding sex education but also facilitates the adoption of home-based sex education practices.

Current parental knowledge, attitudes, and practices (KAP) regarding home-based sex education remain suboptimal. Only 30% of parents achieved a passing knowledge level, with merely 5.07% attaining a good level, reflecting generally low understanding and highlighting an urgent need for enhanced educational initiatives to improve foundational knowledge. Second, although most parents exhibited positive attitude, approximately 70% scored only at a passing level in practice, and fewer than 50% demonstrated good performance. This indicates substantial room for improvement in practical implementation. Finally, the overall KAP consistency rate was approximately 10%, suggesting that few parents effectively translated knowledge into practice.

The current study implemented diverse online intervention approaches, including regular dissemination of educational articles, videos, infographics, and counseling services. In addition, the digital media-based online intervention enables parents to use mobile devices, such as smartphones, to engage in learning during fragmented moments in their daily lives (42). In this study, parents of children showed positive changes in KAP after a 6-month online intervention. Specifically, at the knowledge level, the intervention group led to an improvement of 41.35% in the passing rate and 26.92% in the good-level rate. This advance not only enriched their understanding but also established a foundation for improving attitudes and practices. The intervention also led to significant improvements in parental attitudes toward sex education, reflected by an approximate 10% increase in the attitude pass rate and a 20% rise in the positive rate. Although baseline data indicated that most parents initially supported family sex education implementation (36), the online educational strategies were effective in further enhancing parental enthusiasm for delivering sex education. What’s more, the passing rate of parents’ implementation of family sex education increased by 8.93%, the good rate increased by 15.39%, and the KAP consistency rate increased significantly by 65.58%. According to the TPB (39), parents’ intention to implement family sex education is jointly influenced by their personal attitudes, subjective social norms (environmental influences), and perceived behavioral control (including knowledge and skills related to sex education). In this study, regularly providing parents with diverse learning resources not only significantly enhanced knowledge and skills regarding family sex education but also improved attitudes toward implementing sex education, thereby promoting the actual practice of related behaviors. These findings align with recent international trials, such as a 2025 study in Ethiopia, which demonstrated that empowering parents through structured sexual health education significantly improves parent-adolescent communication and parental self-efficacy across different cultural contexts (43).

Interestingly, the control group’s knowledge scores decreased at the 6-month follow-up. This decline, while seemingly unexpected, aligns with the Ebbinghaus forgetting curve and recent longitudinal evidence. A 2025 study on school-based health interventions (44) demonstrated that health knowledge undergoes steady deterioration over subsequent months in the absence of reinforcement. Similarly, evidence indicates that health-related information is susceptible to rapid decay, often regressing toward the baseline mean within weeks (45). In our study, the control group’s lack of sustained input likely led to this natural regression of fragmented knowledge. Furthermore, the decline may reflect a diminishing social desirability bias, as parents likely reported more realistic, albeit lower, scores at follow-up than at baseline. Ultimately, this deterioration emphasizes the necessity of our 6-month online intervention; it suggests that stable parental capacity for sex education requires the continuous, theory-driven reinforcement provided via our WeChat platform to successfully transition knowledge into long-term memory and practice. This suboptimal baseline is not unique to China; international evidence reveals similar global challenges. For instance, a 2025 study highlighted that even in Western contexts, parents experience profound discomfort and reluctance in using correct anatomical terms with young children, underscoring a universal gap in parental practice, confidence, and comfort levels due to social norms (46).

The shift in parents’ resource preferences further confirms the intervention’s effectiveness. While nearly half of parents initially expressed willingness to learn about sex education online, this proportion exceeded 80% post-intervention, establishing the internet as their preferred information channel. This strong preference for digital media is highly consistent with emerging global trends. A 2025 international qualitative study found that parents urgently recommend “digital-first” resources to overcome their feelings of inadequacy in delivering sex education (47). Furthermore, recent investigations involving European parents confirm a growing acceptance of digital sexuality education tools, emphasizing their utility when content is age-appropriate (48). This is further supported by a 2025 systematic review and meta-analysis which confirmed that web-based sexual health education is globally effective in improving knowledge and behaviors (27). The online education model leverages digital accessibility and abundant resources to deliver early childhood sex education, which overcomes spatiotemporal constraints of traditional approaches, enabling equitable access across regions and socioeconomic backgrounds (49), thereby ultimately supporting sexual health development in young children.

Furthermore, the sensitivity analysis using Generalized Estimating Equations (GEE) confirmed the robustness of our findings against potential cluster-level confounding. The near-zero intra-cluster correlation coefficients indicate that the specific kindergarten environments had a negligible impact on the improvements in parents’ KAP levels. This finding is particularly significant from a public health perspective, as it demonstrates that the efficacy of the TPB-based web intervention is driven by the standardized online content rather than localized school factors. Consequently, this scalable digital model demonstrates promising generalizability, suggesting its potential as a viable and equitable strategy for broader implementation across diverse educational and socioeconomic settings.

This study had several limitations. First, its focus on Luzhou and similar socioeconomic settings may limit generalizability to other regions. Second, the 6-month intervention period was relatively short; longer follow-ups are needed to evaluate sustainability. Third, effect assessments focused primarily on parents; future studies should include children to fully capture intervention impact. Fourth, the difference in questionnaire administration—face-to-face for the control group and online for the intervention group—may have introduced information bias. While face-to-face surveys ensure higher completion rates, online surveys may reduce social desirability bias, potentially leading to differential reporting between groups. Fifth, the sensitivity of topics may have led some parents to conceal information, potentially affecting data authenticity. Although this study implemented measures to ensure confidentiality and minimize bias by providing adequate explanations to obtain parental cooperation, the possibility of reporting bias still remains. Sixth, to avoid model non-convergence issues associated with having only four clusters, we applied Generalized Estimating Equations (GEE) exclusively to our primary continuous KAP scores, retaining Chi-square tests for secondary categorical variables. Although this leaves the cluster effect unadjusted for categorical indicators, the robust GEE results for continuous data strongly validate the intervention’s overall efficacy.

## 5 Conclusion

A TPB-based online intervention effectively enhances parents’ knowledge, attitudes, and practices regarding early childhood sex education while increasing their receptiveness to digital learning. From a public health perspective, this highly scalable and equitable digital model offers a viable strategy to bridge the current family sex education gap. The findings provide evidence-based insights that could inform policymakers and educational institutions in developing future digital platforms for early childhood health promotion, ultimately supporting children’s long-term sexual health and safety.

## Data Availability

The raw data supporting the conclusions of this article will be made available by the authors, without undue reservation.

## References

[1] Joseph JT. Comprehensive sexuality education in the Indian context: challenges and opportunities. Indian J Psychol Med. (2023) 45:292–6. doi: 10.1177/02537176221139566, 37152376 PMC10159571

[2] Lu L Ye Y Zhang W Li H Wang Z Zhang R. Investigation and analysis of demand for early childhood sex education in rural parents, Sichuan. Mod Prev Med. (2021) 48:3120–5. doi: 10.20043/j.cnki.mpm.2021.17.011

[3] Yang J Zhang C Wang Z Lu S Huang N Luo S . Awareness of early childhood sex education and influencing factors among rural kindergarten teachers in Sichuan. Chin J Sch Health. (2021) 42:538–41. doi: 10.16835/j.cnki.1000-9817.2021.04.014

[4] Gubbels J van der Put CE Stams G Assink M. Effective components of school-based prevention programs for child abuse: a meta-analytic review. Clin Child Fam Psychol Rev. (2021) 24:553–78. doi: 10.1007/s10567-021-00353-5, 34086183 PMC8176877

[5] Okeafor CU Okeafor IN Tobin-West CI. Relationship between sexual abuse in childhood and the occurrence of mental illness in adulthood: a matched case–control study in Nigeria. Sex Abus. (2018) 30:438–53. doi: 10.1177/1079063216672172, 27758932

[6] Wen X Ding R Guo C Zheng X. Association between childhood sexual abuse and early sexual debut among Chinese adolescents: the role of sexual and reproductive health education. Front Reprod Health. (2023) 4:909128. doi: 10.3389/frph.2022.909128, 36755898 PMC9900103

[7] Maalouf O Daigneault I Dargan S Mcduff P Frappier JY. Relationship between child sexual abuse, psychiatric disorders and infectious diseases: a matched-cohort study. J Child Sex Abus. (2020) 29:749–68. doi: 10.1080/10538712.2019.1709242, 32045342

[8] Panisch LS Faulkner M Fernandez SB Fava NM. Exploring how trauma is addressed in sexual education interventions for youth: a scoping review. Health Educ Behav. (2020) 47:880–93. doi: 10.1177/1090198120954398, 32900237

[9] Coakley TM Randolph S Shears J Beamon ER Collins P Sides T. Parent–youth communication to reduce at-risk sexual behavior: a systematic literature review. J Hum Behav Soc Environ. (2017) 27:609–24. doi: 10.1080/10911359.2017.1313149, 31485155 PMC6726439

[10] Grossman JM Tracy AJ Charmaraman L Ceder I Erkut S. Protective effects of middle school comprehensive sex education with family involvement. J Sch Health. (2014) 84:739–47. doi: 10.1111/josh.12199, 25274174

[11] Leung H Lin L. Adolescent sexual risk behavior in Hong Kong: prevalence, protective factors, and sex education programs. J Adolesc Health. (2019) 64:S52-8. doi: 10.1016/j.jadohealth.2018.12.007, 31122550

[12] Zhang W Yuan Y. Knowledge, attitudes, and practices of parents toward sexuality education for primary school children in China. Front Psychol. (2023) 14:1096516. doi: 10.3389/fpsyg.2023.1096516, 36818131 PMC9929350

[13] Guilamo-Ramos V Benzekri A Thimm-Kaiser M Dittus P Ruiz Y Cleland CM . A triadic intervention for adolescent sexual health: a randomized clinical trial. Pediatrics. (2020) 145:e20192808. doi: 10.1542/peds.2019-2808, 32345685 PMC7193976

[14] Huang N Luo S Lu S Zhang R Wang Z Yang J . Analysis of the demand and influencing factors for children's sexual education knowledge among rural parents. Chin J Sch Health. (2020) 41:1322–4, 1330. doi: 10.16835/j.cnki.1000-9817.2020.09.012

[15] Guilamo-Ramos V Jaccard J Dittus P Collins S. Parent-adolescent communication about sexual intercourse: an analysis of maternal reluctance to communicate. Health Psychol. (2008) 27:760–9. doi: 10.1037/a0013833, 19025272

[16] Lee Y Florez E Tariman J Mccarter S Riesche L. Factors related to sexual behaviors and sexual education programs for Asian-American adolescents. Appl Nurs Res. (2015) 28:222–8. doi: 10.1016/j.apnr.2015.04.015, 26094879

[17] Lee P Lai H Lin P Kuo S Lin Y Chen S . Effects of a parenting sexual education program for immigrant parents: a cluster randomized trial. Patient Educ Couns. (2020) 103:343–9. doi: 10.1016/j.pec.2019.08.027, 31451362

[18] Koch M Tyson N Bhuinneain GMN Kasliwal A Conry J Sridhar A. FIGO position statement on comprehensive sexuality education. Int J Gynaecol Obstet. (2024) 164:531–5. doi: 10.1002/ijgo.15319, 38219018

[19] Cameron A Smith E Mercer N Sundstrom B. ‘It is our duty:’ understanding parents’ perspectives on reproductive and sexual health education. Sex Educ. (2020) 20:535–51. doi: 10.1080/14681811.2019.1704720, 32952444 PMC7497785

[20] Chi X Hawk ST Winter S Meeus W. The effect of comprehensive sexual education program on sexual health knowledge and sexual attitude among college students in Southwest China. Asia Pac J Public Health. (2015) 27:NP2049–66. doi: 10.1177/1010539513475655, 23417908

[21] Richards SD Mendelson E Flynn G Messina L Bushley D Halpern M . Evaluation of a comprehensive sexuality education program in La Romana, Dominican Republic. Int J Adolesc Med Health. (2021) 33:33. doi: 10.1515/ijamh-2019-0017, 31199763 PMC6986322

[22] Goli S Noroozi M Salehi M. Comparing the effect of two educational interventions on mothers’ awareness, attitude, and self-efficacy regarding sexual health care of educable intellectually disabled adolescent girls: a cluster randomized control trial. Reprod Health. (2021) 18:54. doi: 10.1186/s12978-021-01112-z, 33653361 PMC7923653

[23] Kemigisha E Bruce K Ivanova O Leye E Coene G Ruzaaza GN . Evaluation of a school based comprehensive sexuality education program among very young adolescents in rural Uganda. BMC Public Health. (2019) 19:1393. doi: 10.1186/s12889-019-7805-y, 31660918 PMC6819440

[24] Wang Z Zhao J Jiang H Tian H Yang J Lu S . Effect of sexuality education for parents of preschoolers. Chin J Sch Health. (2022) 43:382–5. doi: 10.16835/j.cnki.1000-9817.2022.03.015

[25] Zhao P Yang L Sa Z Wang X. Propriety, empowerment and compromise: challenges in addressing gender among sex educators in China. Sex Educ. (2020) 20:552–67. doi: 10.1080/14681811.2019.1705779

[26] Scull TM Dodson CV Geller JG Reeder LC Stump KN. A media literacy education approach to high school sexual health education: immediate effects of media aware on adolescents’ media, sexual health, and communication outcomes. J Youth Adolesc. (2022) 51:708–23. doi: 10.1007/s10964-021-01567-0, 35113295 PMC8811737

[27] Guo R Xie H Zhao W Wang J. Web-based sexual and reproductive health education for adolescents aged 10-17 years: a systematic review and meta-analysis. BMJ Paediatr Open. (2025) 9:e003714. doi: 10.1136/bmjpo-2025-003714, 41176330 PMC12581069

[28] China Internet Network Information Center. Statistical Report on the Development Status of China's Internet Network. Beijing: China Internet Network Information Center (2024). Available online at: https://www3.cnnic.cn/n4/2024/0322/c88-10964.html (Accessed January 30, 2026)

[29] Goldfarb ES Lieberman LD. Three decades of research: the case for comprehensive sex education. J Adolesc Health. (2021) 68:13–27. doi: 10.1016/j.jadohealth.2020.07.036, 33059958

[30] Wang F. Promoting healthy growth of children and advancing social sustainable development: an interpretation of 'children and health' in the outline of China's children development (2021-2030). Chin J Women Child Health. (2021) 12:1–4. doi: 10.19757/j.cnki.issn1674-7763.2021.06.001

[31] Xi J Guo K. Keeping pace with the times: the law of the People's Republic of China on the protection of minors. Juv Delinq Prev Res. (2021) 1:21–33. doi: 10.3969/j.issn.2095-3356.2021.02.004

[32] Hua W. On the family education promotion law of the People's Republic of China: its purpose, connotation and implementation. J Nanjing Norm Univ Soc Sci Ed. (2022) 1:58–67. doi: 10.3969/j.issn.1001-4608.2022

[33] Leung H Shek DTL Leung E Shek EYW. Development of contextually-relevant sexuality education: lessons from a comprehensive review of adolescent sexuality education across cultures. Int J Environ Res Public Health. (2019) 16:621. doi: 10.3390/ijerph16040621, 30791604 PMC6406865

[34] Qin S Qin J Su Q Huang T Zhan J Yang X . HIV knowledge, sexual attitudes, and PrEP-eligible behaviors among college students in Southwest China: a cross-sectional study. BMC Infect Dis. (2024) 24:827. doi: 10.1186/s12879-024-09657-7, 39143458 PMC11323675

[35] Wang X Zhang L. Systematic review of sexuality education intervention in the 21st century. Chin J Sch Health. (2021) 42:146–52. doi: 10.16835/j.cnki.1000-9817.2021.01.036

[36] Zhang R Lu L Yu Y Zhou Z Xia H Yan R . Theory of planned behavior-based cross-sectional study of family sex education for preschoolers in China: rural-urban comparative analysis. BMC Public Health. (2025) 25:1130. doi: 10.1186/s12889-025-22365-4, 40128703 PMC11934501

[37] Liao M Lu L Zhou Z Yu Y Song X Zhang X . Analysis of the sources and demand of early childhood sex education knowledge for rural families in Luzhou City. Chin J Hum Sex. (2023) 32:152–6. doi: 10.3969/j.issn.1672-1993.2023.08.039

[38] Bosnjak M Ajzen I Schmidt P. The theory of planned behavior: selected recent advances and applications. Eur J Psychol. (2020) 16:352–6. doi: 10.5964/ejop.v16i3.3107, 33680187 PMC7909498

[39] Ajzen I. The theory of planned behaviour: reactions and reflections. Psychol Health. (2011) 26:1113–27. doi: 10.1080/08870446.2011.613995, 21929476

[40] Parker K Nunns M Xiao Z Ford T Ukoumunne OC. Intracluster correlation coefficients from school-based cluster randomized trials of interventions for improving health outcomes in pupils. J Clin Epidemiol. (2023) 158:18–26. doi: 10.1016/j.jclinepi.2023.03.020, 36997102

[41] Campbell MK Piaggio G Elbourne DR Altman DG Consort Group. Consort 2010 statement: extension to cluster randomised trials. BMJ. (2012) 345:e5661. doi: 10.1136/bmj.e5661 22951546

[42] Song J Lu H. Research on the impact of network fragmentation on mobile learning. J Jilin Prov Inst Educ. (2018) 34:17–20. doi: 10.16083/j.cnki.1671-1580.2018.11.005

[43] Mannion ÁB Conlon C. The vagina problem: a step too far in parent–child sex communication with young children. Sex Educ. (2025) 25:376–89. doi: 10.1080/14681811.2024.2342880

[44] Santos C Azevedo AR Morete NA Maria OC Gradinaru V Carvalhido I . The stroke alert project: a longitudinal evaluation of a school-based stroke knowledge intervention. Cureus. (2025) 17:e99828. doi: 10.7759/cureus.99828, 41573447 PMC12822172

[45] Krishna V Christakis NA. Countering the forgetting of novel health information with “social boosting.”. SSM Popul Health. (2026) 33:101902. doi: 10.1016/j.ssmph.2026.101902, 41737907 PMC12927082

[46] Gutu B Mahimbo A Percival N Demant D. Effect of parent-based sexual health education on parent-adolescent communication and adolescent sexual behavior: a systematic review and meta-analysis. Perspect Sex Reprod Health. (2025) 57:374–422. doi: 10.1111/psrh.70029, 40762112 PMC12421083

[47] Punjani N Scott SD Hussain A. Parents’ information needs and their recommendations for effective sexuality education to children. Front Public Health. (2025) 13:1653924. doi: 10.3389/fpubh.2025.1653924, 41080853 PMC12511028

[48] Hubble TR Carbone L Vandenbosch L Toelen J De Coninck D. Exploring European parents’ attitudes towards the age appropriateness of digital sexuality education for adolescents. Am J Sex Educ. (2025) 20:461–85. doi: 10.1080/15546128.2024.2415304

[49] Jin Z Guo F Wang K Zhang H Cao W Hee J . Effects of an internet-based and teacher-facilitated sexuality education package: a cluster-randomized trial. Children. (2021) 8:885. doi: 10.3390/children8100885, 34682150 PMC8534505

